# Mobile Assistive Application for Blind People in Indoor Navigation

**DOI:** 10.1007/978-3-030-51517-1_36

**Published:** 2020-05-31

**Authors:** Hanen Jabnoun, Mohammad Abu Hashish, Faouzi Benzarti

**Affiliations:** 8 Digital Research Centre of Sfax, Sfax, Tunisia; 9 Institut Mines-Télécom, CNRS, Paris, France; 10 Université de Sherbrooke, Sherbrooke, QC Canada; 11 Digital Research Centre of Sfax, Sfax, Tunisia; 12 University of Sfax, Sfax, Tunisia; 13 ESPRIT, School of Engineering, 2083 Tunis, Tunisia; 14 LR-11-ES17 Signal, Images et Technologies de l’Information (LR-SITI-ENIT), 1002 Tunis Le Belvédère, Tunisia

**Keywords:** Assistive application, Color targets, Camshift algorithm, Android application

## Abstract

Navigation is an important human task that needs the human sense of vision. In this context, recent technologies developments provide technical assistance to support the visually impaired in their daily tasks and improve their quality of life. In this paper, we present a mobile assistive application called “GuiderMoi” that retrieves information about directions using color targets and identifies the next orientation for the visually impaired. In order to avoid the failure in detection and the inaccurate tracking caused by the mobile camera, the proposed method based on the CamShift algorithm aims to introduce better location and identification of color targets. Tests were conduct in natural indoor scene. The results depending on the distance and the angle of view, defined the accurate values to have a highest rate of target recognition. This work has perspectives for this such as implicating the augmented reality and the intelligent navigation based on machine learning and real-time processing.

## Introduction

Vision is a vital human sense that plays a crucial role in the human perception of the environment. However, for the visually impaired, this information is not generally available through external intervention. In fact, to ensure safe and independent mobility, people are usually dependent on external information, planned experience, and existing technology to navigate in indoor environments or in unfamiliar outdoor. In this context, many researchers address the issue of how to enable these individuals to overcome the inability to navigate the environment independently and to understand the visual scene defined by a set of components and characteristics. In this work, we are considering to design a system that provides assistance for the visually impaired to better navigate in both indoor and outdoor environment.

## State of the Art

Recent technological developments provide technical assistance that helps to support visually impaired people in their daily tasks and improves their quality of life. In this context, sensory substitution systems[1–4] are devices that allow information normally acquired by a defective sensory organ and restored to another perceptive modality. For the visually impaired, it consists of transmitting visual information via the auditory or somatosensory system

First, the visuo-tactile substitution devices convert a visual image into tactile information. There are many devices such as the TVSS [5], TDU [6]. These tools are efficient in the recognition of simple forms, the possibility of reading and localization [7].

Moreover, the visuo-auditory substitution devices are based on the transformation of visual image into auditory information. Thanks to auditory system, visually impaired people use sounds for navigation that inform them about the environment and protect them from obstacles. There are many devices such as the Voice [7], PSVA [8], the Vibe [9], and See Colour [10].

These visual substitution systems are using the translation of visual information into another auditory sensory in order to assist visual impaired people. However, existing system needs hard equipment and sometimes the visual impaired should handle it in a backpack.

In addition, new technologies such as mobile phones or smartphones provide technical assistance to support the visually impaired in their daily tasks and aim to improve their quality of life. These devices are equipped with touch screens that ensure better user experience. In fact, they are more adaptable to the visually impaired people using a guiding system for way finding. So that, using a smartphone and its integrated camera, the system is able to detect objects such as the panels [11].

Another technology was presented in literature, which is the Near Field Communication (NFC). NFC is one of the newest technologies in the communication area. It has a rapid progress in mobile devices and an increase number of smartphones equipped with NFC readers. This technology combines identification and interconnection and enables secure communication between electronic devices [12]. In fact, the user has simply to touch the NFC tagged object in order to obtain detailed information.

Besides, there are other visual substitution applications based on voice and haptic replication. The user receives information when entering or sliding the finger on the screen using a screen reader that converts text to speech and ensure reading and navigating through the contents [13].

In addition, haptic is a technology that provides tactile feedback. For tactile-based interfaces [14], haptic can make the user feel and visualize the shape of an element without looking at the screen. It can also provide comments when the finger reaches the limit of an element or button.

The experiments results in literature [15] illustrate the ability of blind and visually impaired people to use assistive mobile application in order to locate the guide signs with an auditory feedback.

## Proposed Method

In order to assist blind and visually impaired people in indoor navigation and facilitate the way finding, we propose a navigation system using mobile application that detects and reads colour targets retrieved through the integrated camera. The developed android application named “GuiderMoi” is mainly used to assist the blind person in indoor environment and buildings.

### General Architecture

**Fig. 1.**
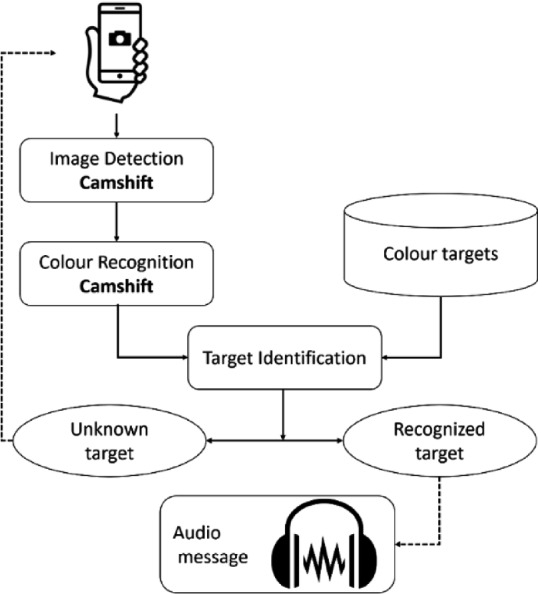
General architecture

The general architecture of the proposed system based on mobile application is shown in Fig. 1.

The colour targets are detected using the Camshift algorithm [16]. Once the colours are determined, a comparison is made with targets in database. If the target is unrecognized, we repeat the operation from the beginning. Otherwise, we continue processing to determine the direction. Finally, once the direction is determined, a vocal notification is launched to notify the user about his next direction.

### Continually Adaptive MeanShift (Camshift) for Target Color Detection

Color targets are a tracking symbols designed to solve the problem of environmental labelling [17]. They are distinctive and difficult to confuse with a typical background clutter and are detectable by a robust algorithm that can work very quickly on a smartphone. Based on the idea of [18], we added the fourth color to detect the last direction.

**Fig. 2.**
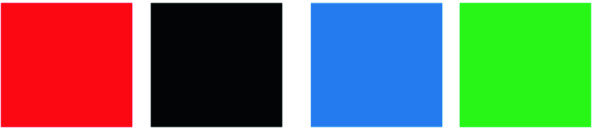
Color targets (Color figure online)

In our proposed system, the colour targets are represented as four squares giving a particular orientation (Fig. 2):

- Red: turn left
- Black: turn right
- Blue: moving forward
- Green: back off

Then, whenever the target is detected, the system provides a vocal directional orientation (for example “turn left”) to guide the visually impaired to its desired destination from its current location.

In the step of color detection of the target, we use color recognition algorithms mainly Camshift.

Furthermore, Camshift (Continually Adaptive MeanShift) is an important algorithm for object tracking based on the color histogram [16]. The algorithm is based on finding the probability distribution map in a search window and iteratively updates the position and size of the window to convergence.

### Camshift Algorithm

The Camshift algorithm uses the meanshift algorithm [16] in a loop varying the size of the window until convergence.

The window in the mean shift is applied with a given size. After convergence, the procedure is re-iterated with a new window, centred on the position found by the mean shift, but with a size depending on the zero order moment of the spatial distribution of the pixels probability previously calculated by the mean shift [16].

The different stages of Cam-shift are as follows:

- Initialize the window *W*: position and size.
- As long as *W* is moved with a certain threshold and the maximum number of iterations is not reached:
  - Apply the mean shift; keep the center $(x_{c} ,y_{c} )$ and the zeo-order moment $M_{oo}$
  - The window W will be centered on $(x_{c} ,y_{c} )$ with the width $w = 2\sqrt {\frac{{M_{00} }}{256}}$ and the height $h = 1.2 w$

Cam-shift is mainly used in the image segmentation. In fact, after convergence of the mean shift, the height of the window is chosen 20% greater than its width, but this choice is arbitrary and can be changed according to the application.

## Results and Discussion

In this section, we present the test conditions and the results of target detection and recognition depending on the distance, the angle of view and the luminosity. In addition, we worked on measuring variables (distance, angle, illumination) because of the sensitivity of mobile application in natural indoor environment conditions.

### Target Detection

**Fig. 3.**
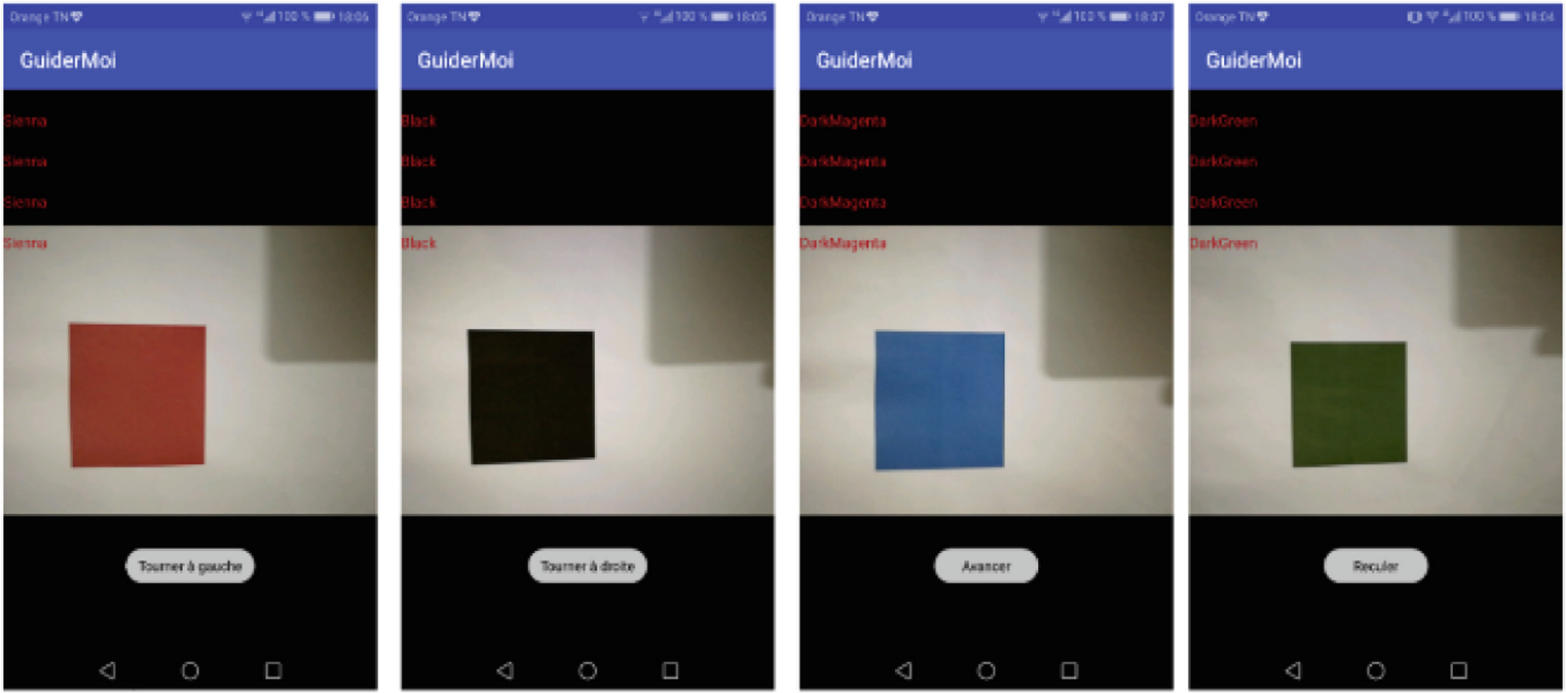
The mobile application (GuiderMoi) detecting targets

The tests are performed in real time in indoor scene. We used the mobile application (GuiderMoi) with variation on the distance, the angle of view and the illumination values (Fig. 3).

#### Distances

First, we choose a reference angle of 90° and we change the distance between the target and the smartphone’s camera calculated in centimetres.

Then, for each selected distance, we detect the target and we repeat the test ten times. Then we calculate the number of successful tests.

In addition, the tests are carried out first in the case of daylight in the case of fluorescent light and finally in the case of incandescent light.

**Fig. 4.**
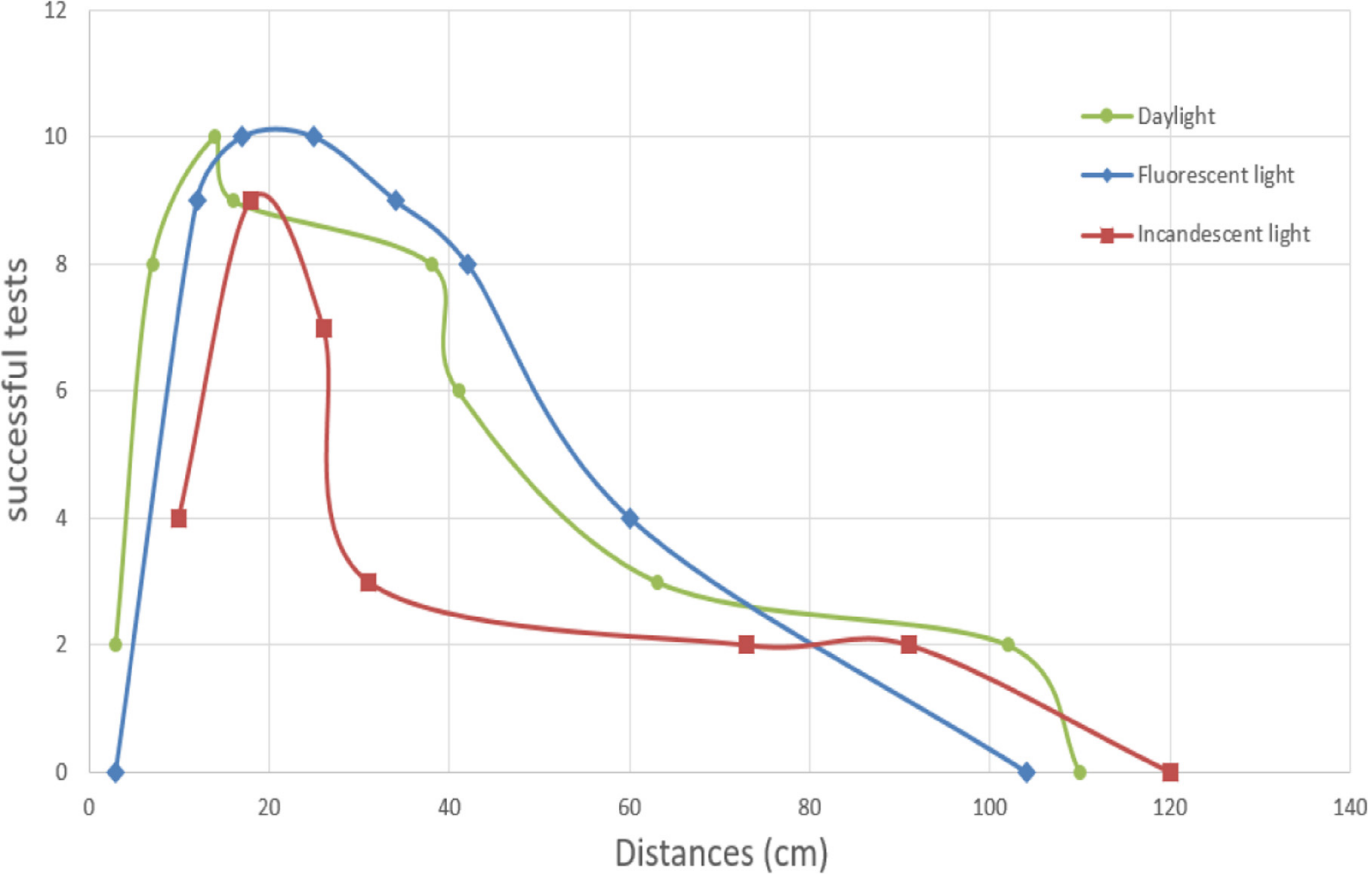
Variation of successful tests by distance in the case of daylight, fluorescent light and incandescent light (Color figure online)

Furthermore, Fig. 4 shows that the target is well detected in the case of daylight but we notice that the detection range increases in the case of fluorescent light and decreases in the case of incandescent light.

#### Angle of vision

In this test, we choose a reference distance of 25 cm and we change the angle between the target and the smartphone camera calculated in degrees.

**Fig. 5.**
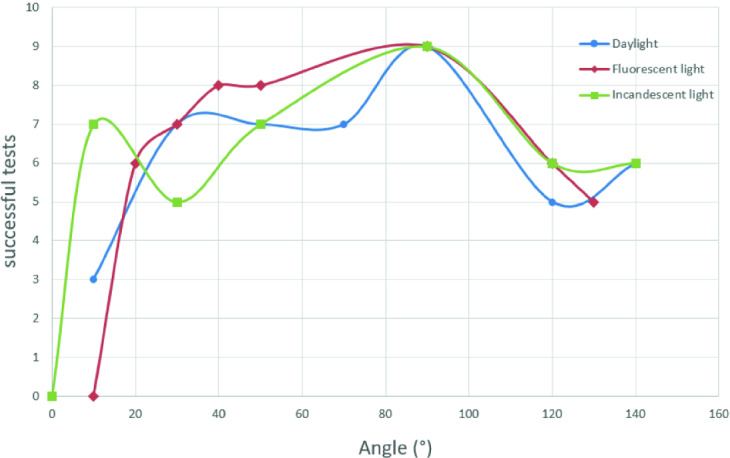
Variation of successful tests by viewing angle case of daylight, fluorescent light and incandescent light (Color figure online)

For each selected angle, we use the application (GuiderMoi) to detect the target and we repeat the test ten times. Then we calculate the number of successful tests. Additionally, the tests are carried out first in the case of daylight, in the case of fluorescent light and finally in the case of incandescent light (Fig. 5).

We notice in Fig. 5 that the detection ranges with respect to daylight increases in the case of fluorescent light and decreases in the case of incandescent light.

### Discussion

First, we tested the mobile application (GuiderMoi) under different conditions mainly distance, angle of view and illumination. We noticed that the colour of the targets was well chosen and their detection is robust especially for the red and the black targets. Then, in the case of illumination, the detection is better in the case of the fluorescent light since it is white and has no influence on the colours.

Moreover, for the distance between the target and the user, the detection is between 15 and 30 cm and the optimal distances are 25 cm. In addition, for the viewing angle, the best position is 90°.

Furthermore, for time processing, the mobile application (GuiderMoi) has the advantage of being in real time. Unfortunately, we have a loss of detection at distance greater than 1 m. In fact, the target becomes smaller and more difficult to identify. At a short distance, we get the reflection of the smartphone on the target that gives false colour detection. Besides, the user has to be exactly in front of the target in order to have a robust detection. We have a total loss of detection at the limits of the target that is to say at an angle of 0° and 180°. The navigation tests were successful in the case of fluorescent light, the user in front of the target with a distance equal to 25 cm.

There are some limitations to our approach. The first is that we are only using the Hue component of the HSV color space. This means that unless the object we are trying to track is not a single shade, then the results will likely be suboptimal.

To remedy this, we can simply extend the code to compute a 2D histogram using both the Hue and Saturation components. However, OpenCV currently does not support 3D histograms in the back projection calculation and Cam-Shift tracking.

The second limitation is tuning the number of bins in the color histogram. This will depend on many aspects, including the application conditions. We need to tune this parameter for our application.

Finally, if we are looking for a more robust tracking solution that can take into account texture and localized features, we should look into keypoints detection, local invariant descriptors (ex. DoG and SIFT), and matching between the sets of keypoints and their corresponding features.

## Conclusion

In this paper, we propose a navigation system based on mobile application “GuiderMoi” to provide assistance for visually impaired in indoor environment. In fact, the mobile application provides information about directions and helps the visually impaired to navigate effectively and independently based on colour targets detection and identification. In future research, it would be interesting to study how we can improve mobile applications using augmented reality and intelligent navigation based on deep learning.
